# Cardiovascular Inflammation

**DOI:** 10.1155/2012/904608

**Published:** 2012-03-19

**Authors:** Masanori Aikawa, Ichiro Manabe, Adrian Chester, Elena Aikawa

**Affiliations:** ^1^Cardiovascular Medicine, Brigham and Women's Hospital, Harvard Medical School, Boston, MA 02115, USA; ^2^Department of Cardiovascular Medicine, The University of Tokyo, Tokyo 113-8655, Japan; ^3^Heart Science Centre, Imperial College London, London 4B9 64H, UK

## 1. Introduction

Westernized countries face a growing burden of cardiovascular diseases including acute complications of coronary atherosclerosis. Cardiovascular diseases often associate with metabolic disorders such as insulin resistance and obesity. Emerging clinical and experimental studies suggest that inflammation is central to the development of these cardiometabolic disorders. A proinflammatory subset of monocytes/macrophages may importantly contribute to pathological processes in cardiometabolic organs. Crosstalks between cardiometabolic organs may enhance systemic and local inflammatory milieu. In addition, accumulating evidence supports a key role for inflammation in the pathogenesis of vascular mineral deposition and calcific aortic valve stenosis. The goal of this special issue is to highlight what is known concerning the inflammatory nature of cardiovascular diseases, such as atherosclerosis and aortic valve calcification, and their association with metabolic disorders. Armed with this understanding, it is hoped that novel therapeutic strategies will aid in the prevention and management of the cardiometabolic syndrome and its complications, and benefit the patients afflicted with calcific aortic valve disease.

## 2. Inflammation and Metabolic Diseases

Obesity, particularly visceral obesity, increases the clinical risk of metabolic and cardiovascular diseases. In addition to its function as a reservoir of lipids, adipose tissue is now known to be an active endocrine organ that produces a variety of “adipokines” and controls energy homeostasis. It has been suggested that the dysregulated production of proinflammatory mediators relative to the production of anti-inflammatory adipokines (e.g., adiponectin) is an important contributor to adverse metabolic and cardiovascular consequences. Recent studies have also demonstrated that the increased secretion of inflammatory mediators seen in obese visceral fat reflects the ongoing chronic inflammation of the adipose tissue, itself. The paper by V. Z. Rocha and E.J. Folco and M. Itoh et al. deal with the molecular mechanisms controlling adipose inflammation and the systemic effects of adipose inflammation on metabolic and cardiovascular diseases. The paper by T. R. Aprahamian and F. Sam focused on adiponectin. M. Furuhashi et al. reviewed the functional involvement of fatty acid-binding proteins in various cells in chronic diseases. These reviews provided molecular insights into development of inflammation in adipose tissue and its propagation to distant tissues that leads to the development of cardiovascular and metabolic diseases.

## 3. Inflammation in Atherosclerosis

Inflammation contributes critically to all stages of atherogenesis. Metabolic disorders such as dyslipidemia promote activation of circulating monocytes and endothelial cells and adhesion of these cell types, leading to accumulation of macrophages. Macrophages then undergo activation and produce proinflammatory cytokines and reactive oxygen species. Oxidative stress activates neighboring cells including endothelial cells and further promotes monocyte recruitment. Such an uncontrolled amplification mechanism represents inflammatory aspects of atherosclerosis. Naturally, many studies have thus addressed whether antioxidants can prevent development of atherosclerosis and its complications and provided unsatisfactory results. In this special issue, F. J. Pashkow overviewed this interesting controversy. Accelerated atherogenesis in patients with autoimmune disease has long been recognized. E. Profumo et al. demonstrated that oxidative stress links these two disorders. Accumulating evidence suggested the causal role of endoplasmic reticulum (ER) stress in apoptosis. But ER stress may also cause various inflammatory disorders including atherosclerosis and metabolic syndrome. T. Gotoh et al. discussed biology of the ER stress pathway in atherogenesis and ischemic cardiac injury. Inflammation not only promotes atherogenesis but also may trigger acute onset of its clinical complication such as coronary thrombosis by reducing mechanical stability of the plaque. D. Segers et al. demonstrated the role of the chemokine CXCL10 in decreased plaque stability as gauged by collagen loss in mouse and human atherosclerotic lesions.

## 4. Inflammation in Cardiac Remodeling

Regardless of the origin, injury to the heart evokes a diverse and complex array of cellular responses involving both cardiomyocytes and nonmuscle cells that initiate and sustain a process of structural remodeling of the myocardium. Remodeling of the myocardium is a key determinant of the clinical course of heart failure. Many of the processes underlying cardiac remodeling have features in common with chronic inflammatory processes. In this issue, the paper by N. Takeda and I. Manabe and Y. Yoshimatsu and T. Watabe reviewed cellular and molecular processes underlying cardiac fibrosis and remodeling. The former paper focuses on the involvement of noncardiomyocytes, highlighting the dynamic cellular interplays in cardiac remodeling. The latter deals with endothelial-mesenchymal transition (EMT), suggesting the endothelial origin of a subset of cardiac fibroblasts. The paper by Y. Feng and W. Chao reviewed the involvement of Toll-like receptors (TLRs) in myocardial responses to infarction and sepsis. TLRs are the major pattern recognition receptors that detect not only the pathogen-associated molecular patterns (PAMPs), but also the damage-associated molecular patterns (DAMPs), including a variety of endogenous molecules. As reviewed in M. Itoh et al., TLRs have also been shown to be important for activation of inflammatory processes in metabolic tissues. These paper provide some of the key ideas in the emerging field of cardiac biology.

## 5. Cardiac Valve Inflammation

It is now widely accepted that inflammatory mechanisms also play a role in development of aortic stenosis. Aortic stenosis shares some risk factors and characteristics with those of atherosclerosis, but remains relatively less amenable to pharmacological intervention. Differences in the aetiology of the different disease process may be a reflection of the unique mechanical environment in which the aortic valve resides. The papers in this special issue that focus on heart valves illustrate the important role inflammatory mediators play in the development, tissue remodeling, and repair of the valve, as well as the initiation and progression of the disease process. The paper by G. J. Mahler and J. T. Butcher examined the role of inflammatory mediators from development to disease. Their paper illustrated how mediators such as TGF-*β*, TNF-*α*, and BMPs play an important role in the self-repair and tissue-remodeling properties that help maintain the durability and strength of the valve. The role of changes in the different types of mechanical force to which the aortic valve is exposed is discussed by K. Balachandran et al. and, more specifically, J. N. Warnock et al. presented new data on the influence of increased levels of pressure in inflammatory gene networks.

We will only then be able to identify targets that might be amenable to molecular or pharmacological modulation after we have gained a comprehensive understanding of the genes expressed, the molecules released, and the signaling pathways activated during the onset and progression of aortic stenosis. While work with animal and human cells and tissue is an important step in this process, development of robust animal models that recapitulates the human disease process will be fundamental to assaying the efficacy of new interventions. In this regard, K. L. Sider and colleagues provided a comprehensive summary of the currently available animal models of aortic stenosis.

## 6. Conclusion

The cardiometabolic syndrome represents a global health burden. An emerging concept suggests that inflammation participates in the pathogenesis of cardiometabolic disorders including atherosclerosis, obesity, insulin resistance, and aortic valve disease. Thus, controlling proinflammatory molecules or pathways may attenuate such diseases. The goal of this thematic series is to highlight what is known concerning the role inflammation plays in the cardiometabolic syndrome and what the therapeutic options are. We believe that this issue will offer updated concepts and help readers develop ideas leading to future investigations and new drug development.

*Masanori Aikawa* *Masanori Aikawa* *Ichiro Manabe* *Ichiro Manabe* *Adrian Chester* *Adrian Chester* *Elena Aikawa* *Elena Aikawa*

